# Health-related quality of life of middle-aged and elderly people with hypertension: A cross-sectional survey from a rural area in China

**DOI:** 10.1371/journal.pone.0246409

**Published:** 2021-02-02

**Authors:** Qi Chen, Li Ran, Mengying Li, Xiaodong Tan

**Affiliations:** Department of Occupational and Environmental Health, School of Health Sciences, Wuhan University, Wuhan, China; Chiang Mai University Faculty of Medicine, THAILAND

## Abstract

**Objective:**

To evaluate health-related quality of life (HRQoL) of middle-aged and elderly people with hypertension in Enshi, China, and to explore the important correlates defining HRQoL.

**Methods:**

From April through July 2018, a self-monitoring intervention program for hypertension control was implemented in a remote area of central China. Participants completed a cross-sectional survey which included demographic characteristics, the Health-related Quality of Life Survey, and the Pittsburgh Sleep Quality Index Survey. Univariate analysis was performed by analysis of variance, and multiple linear regression analysis was used to analyze the influencing factors of HRQoL in middle-aged and elderly hypertensive patients. In the multivariate analysis, the variables with P≤0.05 in the single factor analysis were combined with the professional significance to establish a multiple linear regression model.

**Results:**

Information from 500 participants was available for analysis. Among them, the scores of PCS and MCS was 31.66 ± 9.50 and 41.38 ± 10.69, respectively. Multivariable regression analyses showed that higher education and sleep quality, and moderate physical activity (30 minutes for at least five days a week) had a positive influence on PCS scores. Higher monthly family income (3,000–5,000 ¥) and sleep quality, regular tea-drinking, having 30 minutes of moderate physical activity at least five days a week were positively associated with MCS scores.

**Conclusion:**

The overall HRQoL for rural middle-aged and elderly hypertensive patients in Xuan’en county of Hubei province was poor. Effective relevant measures for the above factors were urgently needed to improve the quality of life for the elderly in rural areas. Awareness of these relevant factors could help health care professionals provide better supportive care.

## Introduction

With the aging and rise of chronic diseases in Chinese population, an increasing number of middle-aged and elderly people suffer from severe health problems [1]. Hypertension (HTN) is considered as a major risk factor for cardiovascular diseases [2]. It is estimated that by 2025, HTN will affect 1.5 billion people around the world, and the yearly number of deaths related to it will increase to 10.4 million [3]. HTN creates a heavy burden in China as well. According to the WHO Study on global AGEing and adult health (SAGE) [4], HTN affects approximately 60% of middle-aged and elderly people in Chinese population, representing a pressing issue in public health.

Health-related quality of life (HRQoL) indicates a person or group’s perceived physical and mental health over time [5–7]. HRQoL is a multidimensional concept of health as well as a widely accepted measure used to assess how the individual’s well-being may be affected over time by a disease, disability, or disorder. It has been extensively investigated in patients with hypertension.

The relationship between hypertension and HRQoL has been examined in many studies. It has been shown that HRQoL levels are lower in hypertensive patients compared to non-hypertensive individuals in a meta-analysis based on 20 observational studies [8]. The lower HRQoL scores in the patients were found to be associated with the frequent occurrence of complications, side effects of anti-hypertensive medications, and, especially difficulty of BP control via prescribed therapeutic procedures [9–11]. Although numerous studies on HRQoL in the rural hypertensive populations have been conducted in China [12,13], no studies for rural middle-aged and elderly patients with hypertension have been reported yet. Moreover, as the prevalence of hypertension increases, many studies have begun to focus on the relationship between hypertension and healthy lifestyles. It was reported that hypertension could be prevented by the adoption of a healthier lifestyle [14], while moderate physical activity could control BP [15] and is positively associated with HRQoL among hypertensive patients [16]. Further, a growing body of evidence has indicated that tea consumption has a protective effect on HTN [17–19], and the habitual tea-drinking of the old is related to better HRQoL [20]. Thus, an unhealthy lifestyle may lead to a reduced HRQoL in hypertensive patients.

In this study, we are primarily focused on examining the current morbidity of hypertension among middle-aged and elderly people in Chinese rural areas. We additionally aimed to identify factors affecting HRQol of hypertensive patients, including socio-demographic characteristics and lifestyle behaviors, allowing health care professionals to provide better supportive care and a reference for further improving the QoL of elderly people with hypertension in rural regions.

## Materials and methods

### Participants and procedures

Data for this study were obtained from a Self-Monitoring Intervention Programme for Hypertension Control in the rural areas of Xuan'en county, Enshi city of Hubei province in China. A population-based and cross-sectional study was conducted from April through July 2018. With the support of the local Health and Family Planning Committee (HFPC), we established a cooperative relationship with the target community prior to participant recruitment and enrollment.

The study recruited participants who met the following criteria: (a) living in the Xuan'en area, (b) aged above 45 years old, (c) having a history of hypertension, receiving anti-hypertensive therapy, or recently diagnosed with hypertension (systolic blood pressure of 140 mm Hg or higher, diastolic blood pressure of 90 mm Hg or higher, or both), (d) being able to possess communication proficiency to fulfill study tasks. The exclusion criteria were listed as follows: (a) with other malignant tumors or serious cognitive impairment, (b) too old to complete the investigation. In addition to providing their personal information, eligible patients were required to accomplish the SF-12 and PSQI (Pittsburgh Sleep Quality Index). For those who could not read or write, the investigator read to the participants without subjective interpretation.

The source of the research objects was the patients with hypertension in the four towns of Zhushan, Xiaoguan, Jiaoyuan, and Wanzhai in Xuan'en County. A cluster random sampling method (with administrative villages as a group) was used to randomly select 22 villages from these 4 towns. Then, we conducted a questionnaire survey among residents who came to the village clinic to measure hypertension, that is, the participants were recruited via convenience sampling. There were 569 potentially eligible participants identified. After eliminating the respondents who had not completed the entire survey or gave invalid responses, 500 valid surveys (87.9%) were retained. The data collection was subjected to quality control measures. The interview groups, each with no less than two local healthcare professionals, conducted the in-person interviews in the respondent’s home based on the structured questionnaires. All the professionals were trained to master questionnaire details, communication capacities, as well as physical fitness test criteria prior to the survey. It took about 20–30 minutes for each participant to accomplish the questionnaire. At the early stage, one supervisor joined each group for properly conducting the interviews. Later on, supervision was routinely carried out in randomly selected groups.

### Study measures

This questionnaire (S1 and S2 Files) is designed by our team members. The survey was tested before it was formally conducted, reviewed, and revised by epidemiology experts to solve the problems found in the pre-survey and determine the final epidemiological survey design plan. Our data is made up of three parts, including socio-demographic and lifestyle behaviors, SF-12, and PSQI.

#### Socio-demographic and lifestyle behaviors

The socio-demographic characteristics comprised gender, age, nationality, marital status, level of education, family monthly income, time since diagnosis, and the number of other chronic diseases. The four age groups were assigned as follows: 45–54, 55–64, 65–74, and 75+. The participants were classified as “married” (including currently married and cohabiting), and “single” (such as never married, divorced, separated, and widowed), based on their marital status, while the following three groups of different education levels were assigned: elementary school or below, junior high school, senior high school or above.

Lifestyle behaviors of participants comprised physical activity, smoking status (smoking was defined as smoking ≥1 cigarette/day at least for six months, while non-smoking referred to never smoking or quit smoking), alcohol intake (drinking was defined as consuming ≥30g alcohol/week in the past year), and tea-drinking status (regular tea-drinking was defined as drinking tea ≥3 times/week for more than 6 months). The above data were also obtained by self-report. “How many days per week do you have 30 minutes of moderate physical activity (such as brisk walking and doing housework)?” was rated on a three-point response scale (1 = 0, 2 = 1–4 days, 3 = at least 5 days).

#### The Chinese version of SF-12

The second part is the SF-12, which was used to determine the HRQoL [21]. As a shorter and viable form of the SF-36, SF-12 has been successfully utilized to evaluate HRQoL in the cohorts of healthy and chronically ill individuals among different countries [22–25]. In the SF-12 questionnaire, weighted subscales were employed to score the Physical Component Summary (PCS) and Mental Component Summary (MCS). PCS includes general health, physical functioning, role-physical, and bodily pain, while MCS comprises vitality, social functioning, role-emotional, and mental health. A higher score on the questionnaire is indicative of better HRQoL. The SF-12 proves to be a generic questionnaire widely used for the assessment of HRQoL [17]. In addition, its reliability and validity of the scale for determining the QoL of middle-aged and elderly people with hypertension has been confirmed. The Cronbach’s alpha value in this study was 0.801.

#### The PSQI

The third part is PSQI. PSQI scale [26], a well-validated instrument widely used for assessing sleep quality [27–30], was utilized to determine sleep quality in the study. The PSQI consists of seven sleep score factors including subjective sleep quality, sleep latency, sleep duration, habitual sleep efficiency, sleep disturbances, use of sleeping medication, and daytime dysfunction, each of which is scored using a scale running between 0 and 3. And a higher score means a worse factor. The cumulative scores of each factor constitute the total factor score of PSQI, ranging from 0 to 21 points. According to the total score of PSQI, this section was classified into three levels: ≤ 4 (sleep well), 5~6 (sleep general), and ≥ 7 (poor sleep or a sleep disorder). The reliability of PSQI in this study evaluated using the Cronbach alpha was 0.703.

## Ethics statement

The present study was approved by the Research Ethics Committees of Wuhan University (2018-1602000-03-02) and the local institutional review board of Xuan’en, Hubei. All procedures involving human participants followed the ethical standards of the institutional research committee and the guidelines of the Declaration of Helsinki. All participants provided written informed consent. No human or animal subjects, human cell lines, or human tissues were performed.

## Statistical analysis

A database was generated using Epidata3.1 software, and all data were double entered and verified. The SF-12 and PSQI instruments were scored according to the respective scoring methods. Continuous variables and categorical data were expressed as mean ± SD and percentage, respectively.

To identify HRQoL determinants, first, the effects of participants' characteristics on each SF-12 scale/item were examined. In this case, one-way analysis of variance or the nonparametric Kruskal-Wallis test was performed on categorical data, depending on how the variables were distributed. Pearson correlation analysis was carried out to determine the relationship between continuous variables and QoL scores.

Then, variables with statistical significance were entered into a multiple linear regression model. The regression analysis was performed on dichotomous variables to simplify their correlations. After the assumptions on independence and distribution of the residuals as well as multicollinearity were checked, the multiple linear stepwise regression method was employed to explore the independent associations among socio-demographic characteristics, lifestyle behaviors, sleep quality, and scores of PCS or MCS. SPSS software (version 22.0) was used to undertake the statistical analysis. Statistically, values of two-sided p ≤0.05 indicated a significant difference.

## Results

### General data

**Table 1** presented the basic information of the participants in this study. A total of 500 participants were comprised of 218 (43.60%) males and 282 (56.40%) females. All participants were aged 68.92±9.43 years, the minimum age was 45 and the maximum age was 97, with 70.72 ± 9.27 years for males and 67.52 ± 9.33 years for females. About half (50.20%) of the participants were Tujia. Education level of the large majority (82.60%) of the participants was classified as elementary school or below, and 72.60% were married. The family monthly income was categorized as < ¥3,000, ¥3,000–5,000, and > ¥5,000, and 87.60% of the participants had a monthly family income of less than 3,000 Yuan. For all participants, the percentages of smoking, drinking, and regular tea-drinking were 24.40%, 20.60%, and 59.80%, respectively.

**Table 1:** Demographic and clinical characteristics (N = 500).

Characteristics	Number	%
Gender		
Male	218	43.60
Female	282	56.40
Age		
45~54	47	11.80
55~64	92	21.60
65~74	217	40.40
75+	144	26.20
Nationality		
Han	160	32.00
Tujia	251	50.20
Other minorities	89	17.80
Education level		
Elementary school or below	413	82.60
Middle school	66	13.20
High school or above	21	4.20
Marital status		
Single	137	27.40
Married	363	72.60
Years of hypertension		
0–3 years	161	32.20
4–6 years	113	22.60
≥7 years	226	45.20
Number of comorbid diseases		
0	225	45.00
1	189	37.80
≥2	86	17.20
Family monthly income(yuan)		
<3,000	438	87.60
3,000–5,000	39	7.80
>5,000	23	4.60
Smoking status		
Yes	122	24.40
No	378	75.60
Alcohol intake		
Yes	103	20.60
No	397	79.40
Regular tea-drinking		
Yes	299	59.80
No	201	40.20
Moderate physical activity per week		
None	113	22.60
1–4 days	63	12.60
≥5 days	324	64.80
Sleep quality status		
Badly	257	51.40
General	118	23.60
Well	125	25.00

### Overall HRQoL

SF-12 scale and summary scores of 500 middle-aged and elderly individuals in the region (Mean ± SD) were described in **Table 2**. The mean PCS and MCS of HRQoL in this population were 31.66±9.50 and 41.38±10.69, respectively. Scores for physical functioning, role-physical, bodily pain, and general health were 36.74±12.60, 23.00±4.14, 32.50±12.26, and 32.74±12.70, respectively, while vitality, role-emotional, social functioning, and mental health scores were 45.29±13.81, 47.62±12.28, 17.55±5.44, and 47.66±13.89, respectively.

**Table 2:** QoL scores of 500 rural middle-aged and elderly patients with hypertension in Xuan’en County (M ± SD).

Scale	Scores
Physical functioning	36.74±12.60
Role-physical	23.00±4.14
Bodily pain	32.50±12.26
General health	32.74±12.70
Vitality	45.29±13.81
Role-emotional	47.62±12.28
Social functioning	17.55±5.44
Mental health	47.66±13.89
PCS	31.66±9.50
MCS	41.38±10.69

PCS: Physical component summary; MCS: Mental component summary.

The PCS and MCS scores of hypertensive patients (M ± SD) were described in **Table 3**. Significant differences in PCS can be found for education level, the number of comorbid diseases, sleep quality status, regular tea-drinking, and physical activity frequency. The MCS was affected by years of hypertension, the number of comorbid diseases, sleep quality status, regular tea-drinking, and physical activity frequency. Besides, there were no statistically significant differences in distinct dimensions, PCS and MCS among the groups of different gender, age, nationality, marital status, smoking status, and alcohol intake.

**Table 3:** Univariate analysis of factors influencing the QoL in 500 rural middle-aged and elderly patients with hypertension in Xuan'en County (Mean±SD).

Variable		PCS		MCS	
		Mean±SD	p-value	Mean±SD	p-value
Gender			0.711		0.092
	Male	31.49±9.60		42.30±10.67	
	Female	31.80±9.43		40.67±10.67	
Age group (in years)			0.131		0.696
	45–54	33.67±10.47		41.86±12.09	
	55–64	31.65±8.98		41.37±10.17	
	65–74	32.15±9.73		40.79±11.10	
	75+	30.30±9.03		42.12±9.92	
Nationality			0.769		0.173
	Han	31.96±9.82		40.73±10.08	
	Tujia	31.36±9.01		42.25±10.94	
	Other minorities	32.00±10.31		40.10±10.95	
Education level			0.013		0.068
	Elementary school or below	31.22±9.47		40.93±10.81	
	Middle school	34.84±9.36		42.83±10.02	
	High school or above	30.53±8.90		45.70±9.33	
Marital status			0.708		0.453
	Single	31.41±10.35		40.80±11.06	
	Married	31.76±9.17		41.60±10.55	
Years of hypertension			0.153		0.047
	≤3	32.80±9.58		42.62±0.48	
	4–6	31.26±9.67		42.19±10.11	
	≥7	30.96±9.23		40.08±11.03	
Number of comorbid diseases			<0.0001		<0.0001
	0	33.32±9.96		43.87±10.45	
	1	31.01±9.28		39.79±10.13	
	≥2	28.78±7.81		38.39±11.16	
Family monthly income (yuan)			0.218		0.004
	<3,000	31.59±9.30		40.79±10.66	
	3,000–5,000	30.65±9.43		44.86±10.09	
	>5,000	34.84±12.66		46.68±10.06	
Sleep quality status			<0.0001		<0.0001
	Badly	29.02±8.81		38.60±10.93	
	General	32.74±9.39		43.37±9.94	
	Well	36.08±9.19		45.24±9.24	
Smoking status			0.821		0.435
	Yes	31.50±9.63		42.04±10.42	
	No	31.72±9.47		41.17±10.78	
Alcohol intake			0.180		0.103
	Yes	32.78±10.09		42.91±10.20	
	No	31.37±9.33		40.99±10.79	
Regular tea-drinking			0.016		0.004
	Yes	32.50±9.64		42.51±10.21	
	No	30.42±9.17		39.70±11.18	
Moderate physical activity per week			<0.0001		<0.0001
	None	29.62±10.02		38.54±10.87	
	1–4 days	28.61±7.81		38.56±9.78	
	>5 days	32.97±9.37		42.92±10.51	

PCS: Physical component summary; MCS: Mental component summary.

### Correlations of HRQoL with socio-demographic and lifestyle variables

The linear regression assumptions were met for the variables. And Linearity, homogeneity of variance, and normality of the residuals were validated in the models. The d-values for the Durbin–Watson test were 1.87 for PCS and 2.14 for MCS. The VIFs were <10, indicative of the absence of multicollinearity in the models.

Next, we undertook multivariable analyses to identify those variables having an independent significant impact on HRQoL. PCS and MCS were identified as dependent variables in the models, while independent variables included education (elementary school and below as reference), years of hypertension (≥7 as reference), the number of comorbid diseases (0 as reference), monthly family income (<3,000 ¥ as reference), sleep quality (sleep badly as reference), regular tea-drinking (no as reference) and 30 minutes of moderate physical activity per week (none as reference).

As shown in **Table 4**, higher education and sleep quality, and moderate physical activity (30 minutes for at least five days a week) had a positive influence on PCS scores, while people with an education level of middle school had higher scores than those with elementary school and below. Moreover, higher monthly family income (3,000–5,000 ¥) and sleep quality, regular tea-drinking, and moderate physical activity were found to be independently correlated with higher MCS scores. Years of hypertension and the number of comorbid diseases appeared not to be significantly correlated with HRQoL.

**Table 4:** Multi-factor analysis of factors influencing the quality of life of rural middle-aged and elderly people in Xuan'en County.

Variable		PCS		MCS	
		Coeff.	p-value	Coeff.	p-value
Education level (Elementary school or below as reference)					
	Middle school	3.056	0.011	1.106	0.416
	High school and above	-1.698	0.418	2.671	0.262
Years of hypertension (≥7 as reference)					
	≤3	1.058	0.258	1.519	0.152
	4~6	-0.331	0.750	1.044	0.374
Number of comorbid diseases (0 as reference)					
	1	-1.376	0.267	-2.340	0.096
	≥2	-1.310	0.582	-1.011	0.707
Family monthly income (<3,000RMB as reference)					
	3,000–5,000	-1.147	0.448	3.288	0.046
	>5,000	1.889	0.344	2.824	0.212
Sleep quality (Sleep badly as reference)					
	Sleep general	3.276	0.001	4.156	0.000
	Sleep well	6.343	0.000	5.325	0.000
Regular tea-drinking (No as reference)		1.282	0.120	1.965	0.036
Moderate physical activity per week (None as reference)					
	1–4	-1.238	0.382	0.007	0.996
	>4	2.003	0.044	3.107	0.006

PCS: Physical component summary; MCS: Mental component summary.

## Discussion

Hypertensive patients are known to present a lower HRQoL compared with the normotensives, however, the present study was the first to evaluate HRQoL of middle-aged and elderly hypertensives in Chinese rural areas, and identify the important variables correlated with HRQoL. The SF-12 was the most frequently used instrument for specifically determining HRQoL among patients with hypertension. Here, middle-aged and elderly patients in Xuan’en County generally showed PCS and MCS scores below normal limits, indicating a low HRQoL [31].

As seen from our study, education level is significantly associated with PCS in multivariable analyses—hypertensive patients with a higher education level have better HRQoL, being consistent with previous research [32,33]. Although self-care awareness of middle-aged and elderly people is relatively weak, patients with a higher education level perform better in psychological adjustment and social adaptability than those with lower education. Moreover, they can grasp their physical status better so as to seek more appropriate ways to improve individual’s condition. Previous data indicated that comorbid diseases might affect the severity of the disease and physical discomfort, leading to worse health status [34]. However, in our study, HRQoL was not influenced by the number of comorbid diseases. This discordance might be explained because of the participants' differences.

Patients with regular tea-drinking had higher MCS scores than those not, which is in agreement with previous research [20], however, it showed no significant effect on PCS scores in our study. Although consumption of no less than five cups of black tea per day was shown to be correlated with a lower blood pressure in some study [35], epidemiological investigations in determining the effect of regular tea-drinking on hypertension remain inconclusive [36]. This study was limited by the fact that we had not considered tea consumption in the work. In addition, monthly family income was found to be significantly associated with MCS scores in this study, and a certain economic foundation has been proved psychological protective for residents. This may be because low-income people tend to have higher healthcare needs, but they do not have enough ability to pay for medical services [13]. The discomfort caused by lack of medical services exacerbates people's dissatisfaction with their own health, which in turn has a negative impact on people's HRQoL. Thus, given that the middle-aged and elderly people, especially those in the rural area, have poor less economic independence and low labor capacity, many research questions in this series, such as the elderly's diet and medical treatment, need to be further addressed.

Notably, we found that sleep quality and physical exercise played an important role in determining HRQoL of hypertensive patients. Previous studies have shown that sleep is an important determinant of health as it is closely related to mortality [37] and morbidity [38]. An association of a high global PSQI score with elevated hypertension morbidity was also reported in a rural Chinese cohort [39]. As is acknowledged, poor sleep quality may contribute decreased attention and memory loss, while insufficient sleep time or long-term low-quality sleep can affect the body's immunity, making people prone to illness and leading to a decline in quality of life. To cope with it, we suggest middle-aged and elderly hypertensive patients incense and foot bathing to improve sleep quality. In addition, this study revealed that hypertensive patients exercised more than 4 times a week had higher PCS scores than those who did not exercise, whereas no significant difference in PCS scores was found between the patients who exercised more than 4 times a week and those who exercised 1–4 times a week. Regular physical activity prevents hypertension and is also a basic part of the treatment of hypertension [40]. This suggests that middle-aged and elderly hypertensive patients should exercise more, and exercising more than four times a week can effectively improve their quality of life.

The results obtained in this study provide new insights into how to untangle the complex relationships among socio-demographic characteristics, lifestyle habits, sleep quality, and PCS and MCS scores for optimizing HRQoL of hypertensive patients. Despite these interesting findings, some of the limitations in this work need to be discussed. First, this study may not fully represent the situation in all regions of the country due to the fact that sampling was limited to the area of Xuan’en County, Hubei Province. Xuan’en County is a poverty-stricken county with relatively poor educational resources. More than 70% of the participants only had a primary school level or below, and the family monthly income was less than 3,000 yuan. Second, given that the study is a population-based survey where socio-demographic and lifestyle behaviors were self-reported, recall bias may happen regardless of the data collection by in-person interviews. Third, the blood pressure of the study subjects was not graded. Lastly, this cross-sectional survey could not draw causal inferences concerning the relationship between targeting variables and HRQoL. Instead, bidirectional effects may occur in this situation. Further longitudinal studies are needed to examine the causation of observed association and better tailor intervention for middle-aged and elderly individuals with hypertension.

## Conclusions

This cross-sectional survey found that middle-aged and elderly people with hypertension in Xuan’En county had a poor HRQoL. Overall, improving HRQoL of middle-aged and elderly people in rural areas has a long way to go. At the root of it, it is necessary to develop the economy of rural areas and improve the local economic level. Equally importantly, health education for hypertensive patients on improving the prevention of hypertension as well as developing healthy habits including regular physical exercise needs to be promoted. Moreover, we recommend that while the management and monitoring of hypertension for the elderly implemented by the health administration should be strengthened, the free medical examination under public health programs is essential for the population.

## Supporting information

S1 File Original study questionnaire. (DOC)

S2 File English study questionnaire. (DOCX)

S1 Data (XLSX)

## Abbreviations

HRQoL: Health related quality of life
QoL: Quality of life
PCS: Physical component summary
MCS: Mental component summary
PSQI: Pittsburgh Sleep Quality Index
