# Supplementary material for: Health-related quality of life of middle-aged and elderly people with hypertension: A cross-sectional survey from a rural area in China
Source: PLoS One. 2021 Feb 2;16(2):e0246409. doi: 10.1371/journal.pone.0246409 (PMC7853450; doi:10.1371/journal.pone.0246409)
Supplement: S1 File — (DOC) [file pone.0246409.s001.doc]

**恩施市宣恩县居民高血压流行病学调查问卷**

问卷编号： 

**第一部分 基本资料**

**A1**性别：①男 ②女

**A2**出生年月： 年 月

**A3**您的民族：①汉族 ②土家族 ③其他少数民族

**A4**婚姻状况：①未婚 ②已婚 ③离异 ④丧偶

**A5**文化程度： ①小学及以下 ②初中 ③高中及以上

**A6**您诊断为高血压的年限有: ①0-3年 ②4-6年 ③≥7年

**A7**您的家庭月收入是 元？

①＜3000元 ②3000-5000元 ③＞5000元

**A8**您目前是否患有下列疾病？(多选项)

①糖尿病 ②心脏病 ③高血脂 ④肾脏疾病 ⑤关节炎/风湿病

⑥脑卒中 ⑦眼底出血 ⑧慢支或慢阻肺

**第二部分 生活方式**

**B1**您平均每周有几天进行30分钟的**适度体育活动**(比如快走和做家务)？

①＜1天； ②1~4天； ③＞4天

**B2**您**吸烟**吗？ ①吸烟（在过去的六个月中，每天吸烟≥1支） ②不吸烟（从不吸烟或戒烟）

**B3**您**喝酒**吗？①喝酒（在过去的一年中，每周饮酒≥30克） ②不喝酒

**B4** 您**喝茶**吗？ ①喝茶（在过去的六个月中，每周喝茶≥3次） ②不喝茶

**第三部分 生活质量**

**C1**总括来说，您认为您现在的健康状况是：（1）非常好 （2）很好 （3）好 （4）一般 （5）差
**C2**进行中等强度活动时，您是否会受到健康状况的限制，比如移动桌子，推动吸尘器，打保龄球，打高尔夫球？如果受到限制，受限程度有多大？

（1）有好大限制 (2)有点限制 (3)没有任何限制

**C3**您的健康状况对您爬楼梯的限制程度有多大？
（1）有好大限制 (2)有点限制 (3)没有任何限制

**C4**在过去4个星期，您会否因为身体健康的原因而令您在工作或日常活动中，实际做完的或者完成的比想做的少？ (1)会 (2)不会

**C5**在过去4个星期，您会否因为身体健康的原因而使您的工作或活动受到限制？

(1)会 (2)不会

**C6**在过去4个星期，您会否因为情绪方面的原因(比如感到沮丧或焦虑)，而令您在工作或日常活动中实际做完或完成的比想做的少? (1)会 (2)不会

**C7**在过去4个星期，您会否因为情绪方面的原因(比如感到沮丧或焦虑)而您的工作或活动受到限制？

(1)会 (2)不会

**C8**在过去4个星期里，您**身体上的疼痛对您的日常工作(包括上班和家务)有**？

(1)完全没有影响 (2)有很少影响 (3)有些影响 (4)有较大影响

(5)有非常大的影响 (6)不适用

**C9**在过去4个星期，您有**多少时间感到心平气和**？

(1)常常 (2)大部分时间 (3)很多时间 (4)间中

(5)只有很少时间 (6)从来没有

**C10**在过去4个星期，您有多少时间**感到精力充足**？

(1)常常 (2)大部分时间 (3)很多时间 (4)间中

(5)偶尔一次半次 (6)从来没有

**C11**在过去4个星期里，您有多少时间**觉得心情不好，闷闷不乐或沮丧**？

(1)常常 (2)大部分时间 (3)很多时间 (4)间中

(5)偶尔一次半次 (6)从来没有

**C12**在过去4个星期，有多少时间**由于您身体健康或情绪问题而妨碍您的社交活动**（比如探亲、访友等）？

(1)常常 (2)大部分时间 (3)很多时间 (4)间中

(5)偶尔一次半次 (6)从来没有

**第四部分 睡眠质量**

| **D1**近1个月，您晚上上床睡觉的时间通常是 点钟（24小时制） | | | | |
| --- | --- | --- | --- | --- |
| **D2**近1个月，您从上床到入睡通常需要 分钟 | | | | |
| **D3**近1个月，您通常起床时间是 点钟（24小时制） | | | | |
| **D4**近1个月，您每夜通常实际睡眠时间 小时 | | | | |
| **D5**近1个月，您有没有因下列情况而影响睡眠，请在相应的□中打“√” | | | | |
|  | 无 | 不足1次/周 | 1-2次/周 | 3次或以上/周 |
| **D5a**入睡困难(30分钟内不能入睡) | □ | □ | □ | □ |
| **D5b**夜间易醒或早醒 | □ | □ | □ | □ |
| **D5c**夜间去厕所 | □ | □ | □ | □ |
| **D5d**呼吸不畅 | □ | □ | □ | □ |
| **D5e**大声咳嗽或鼾声高 | □ | □ | □ | □ |
| **D5f**感觉冷 | □ | □ | □ | □ |
| **D5g**感觉热 | □ | □ | □ | □ |
| **D5h**做噩梦 | □ | □ | □ | □ |
| **D5i**疼痛不适 | □ | □ | □ | □ |
| **D5j**其他影响睡眠的事情 | □ | □ | □ | □ |
| **D6**近1个月您的睡眠质量如何？ ①很好 ②较好 ③较差 ④很差 | | | | |
| **D7**近1个月您是否经常使用催眠药物才能入睡？  ①无 ②不足1次/周 ③1-2次/周 ④3次或以上/周 | | | | |
| **D8**近1个月您是否感到困倦？  ①无 ②不足1次/周 ③1-2次/周 ④3次或以上/周 | | | | |
| **D9**近1个月您是否感到精力不足？  ①没有 ②偶尔有 ③有时有 ④经常有 | | | | |

**参与者签字：** 您的联系电话： **研究者签字：**
