# Supplementary material for: Health-related quality of life of middle-aged and elderly people with hypertension: A cross-sectional survey from a rural area in China
Source: PLoS One. 2021 Feb 2;16(2):e0246409. doi: 10.1371/journal.pone.0246409 (PMC7853450; doi:10.1371/journal.pone.0246409)
Supplement: S2 File — (DOCX) [file pone.0246409.s002.docx]

**Questionnaire on the epidemiology of hypertension among residents in Xuan 'en County of Enshi city**

**Part One Basic Information**

A1. Your gender: [single choice] *

①Male ②female

A2. Date of birth: [fill in the blanks]*

______ Year ______ Month

A3. Your national: [single choice] *

①Han nationality ②Tujia nationality ③Other minority nationalities

A4. Your marital status: [single choice] *

①Unmarried ②Married ③Divorced ④Widowed

A5. Your level of education [single choice] *

①Elementary school and below ② Middle school ③High school and above

A6. How long have you been diagnosed with hypertension? [single choice] *

①0-3 years ② 4-6 years ③≥7 years

A7. Your monthly family income?

①＜3,000 yuan ②3,000-5,000 yuan ③＞5,000 yuan

A8. Do you currently suffer from the following diseases? (Multiple options)

①Diabetes ②Heart disease ③Hyperlipidemia ④Kidney disease ⑤Arthritis/rheumatism

⑥Stroke ⑦Fundus hemorrhage ⑧Chronic bronchitis or COPD

**Part Two Lifestyle**

B1. On average, how many days do you do 30 minutes of moderate physical activity per week (such as brisk walking and housework)?

①＜1 day

②1~4 days

③＞4 days

B2. Do you smoke?

①Smoking (≥1 cigarette/day in the past six months)

②Never smoking (never smoking or quitting)

B3. Do you drink?

①Yes (≥30g/week in the past year)

②No

B4. Do you drink tea?

①Yes (≥3 times/week in the past six months)

②No

**Part Three Quality of Life**

**This survey asks for your views about your health. This information will help keep track of how you feel and how well you are able to do your usual activities. Please answer every question by marking one box. If you are unsure about how to answer, please give the best answer you can.**

C1. In general, your current health status is: [single choice] *

（1）Excellent （2）Very good （3）Good （4）Fair （5）Poor

**The following questions are about activities you might do during a typical day. Does your health now limit you in these activities? If so, how much?**

C2. Moderate activities, such as moving a table, pushing a vacuum clearner, bowling, or playing golf [single choice] *

(1)Yes, limited a lot

(2)Yes, limited a little

(3)No, not limited at all

C3. Climbing several flights of stairs [single choice] *

(1)Yes, limited a lot

(2)Yes, limited a little

(3)No, not limited at all

**During the PAST FOUR WEEKS, have you had any of the following problems with your work or other regular daily activities as a result of your physical health?**

C4. Accomplished less than you would like [single choice] *

(1)Yes

(2)No

C5. Were limited in the kind of work or other activites [single choice] *

(1)Yes

(2)No

**During the PAST FOUR WEEKS, have you had any of the following problems with your work or other regular daily activities as a result of any emotional problems (such as feeling depressed or anxious)?**

C6. Accomplished less than you would like [single choice] *

(1)Yes

(2)No

C7. Didn't do work or other activities as carefully as usual [single choice] *

(1)Yes

(2)No

C8. **During the PAST FOUR WEEKS,** how much did pain interfere with your normal work (including both work outside the home and housework)? [single choice] *

(1)Not at all

(2)A little bit

(3)Moderately

(4)Quite a bit

(5)Extremely

**These questions are about how you feel and how things have been with you during the PAST FOUR WEEKS. For each question, please give the one answer that comes closest to the way you have been feeling. How much of the time during the past week?**

C9. Have you felt calm and peaceful? [single choice] *

(1) All of the Time

(2) Most of the Time

(3) A Good Bit of the Time

(4) Some of the Time

(5) A little of the Time

(6) None of the Time

C10. Did you have a lot of energy? [single choice] *

(1) All of the Time

(2) Most of the Time

(3) A Good Bit of the Time

(4) Some of the Time

(5) A little of the Time

(6) None of the Time

C11. Have you felt downhearted and depressed? [single choice] *

(1) All of the Time

(2) Most of the Time

(3) A Good Bit of the Time

(4) Some of the Time

(5) A little of the Time

(6) None of the Time

C12. **During the PAST FOUR WEEKS,** how much of the time has your physical health or emotional problems interfered with your social activities (like visiting with friends, relatives, etc.)? [single choice] *

(1) All of the Time

(2) Most of the Time

(3) Some of the Time

(4) A little of the Time

(5) None of the Time

**Part IV Sleep quality**

| **D1** In the past month, the time you go to bed at night is usually o'clock (24-hour clock) | | | | |
| --- | --- | --- | --- | --- |
| **D2** In the past month, it usually takes minutes from going to bed to falling asleep | | | | |
| **D3** In the past month, you usually wake up at o'clock (24-hour clock) | | | | |
| **D4** In the past month, you usually sleep hours per night | | | | |
| **D5** In the past month, have you affected your sleep due to the following conditions, please tick “√” in the corresponding □ | | | | |
|  | None | ＜once/week | 1-2 times/week | ≥3 times/week |
| **D5a** Difficulty in falling asleep (cannot fall asleep within 30 minutes) | □ | □ | □ | □ |
| **D5b** Wake up easily or early at night | □ | □ | □ | □ |
| **D5c** Go to the bathroom at night | □ | □ | □ | □ |
| **D5d** Breathless | □ | □ | □ | □ |
| **D5e** Loud cough or high snoring | □ | □ | □ | □ |
| **D5f** Feeling cold | □ | □ | □ | □ |
| **D5g** Feeling hot | □ | □ | □ | □ |
| **D5h** Have nightmares | □ | □ | □ | □ |
| **D5i** Pain and discomfort | □ | □ | □ | □ |
| **D5j** Other things that affect sleep | □ | □ | □ | □ |
| **D6** How was your sleep quality in the past month?  ① very good ② good ③ poor ④ very poor | | | | |
| **D7** Have you often used hypnotics to fall asleep in the past month?  ①None ②＜once/week ③1-2 times/week ④≥3 times/week | | | | |
| **D8** Have you felt sleepy in the past month?  ①None ②＜once/week ③1-2 times/week ④≥3 times/week | | | | |
| **D9** Have you felt less energetic in the past month?  ①No ②Occasionally ③Sometimes ④Always | | | | |

Participant's signature:

Your phone number:

Investigator's signature:
